# Modeling the LFP footprint of unitary thalamic inputs to sensory cortex

**DOI:** 10.1186/1471-2202-12-S1-P86

**Published:** 2011-07-18

**Authors:** Espen Hagen, Janne C Fossum, Klas H Pettersen, Jose-Manuel Alonso, Harvey A Swadlow, Gaute T Einevoll

**Affiliations:** 1 Dept. of Mathematical Sciences & Technology, Norwegian Univ. Life Sciences, Ås, NO-1432, Norway; 2 Dept. of Biological Sciences, SUNY College of Optometry, NY 10036, USA; 3 Dept. of Psychology, University of Connecticut, Storrs, CT 06269, USA

## 

The depth-resolved synaptic local-field potential (LFP) footprint in sensory cortex following firing in individual thalamic projection neurons can be accurately measured by averaging cortical multielectrode (ME) LFP signals over thousands of spontaneous thalamic firing events [1,2]. This spike-triggered LFP method offers a unique window into the thalamocortical connection. However, the interpretation of the detailed spatiotemporal profile of this LFP footprint is not trivial as the LFP signal reflects a weighted sum over contributions from all dendritic transmembrane currents located in the vicinity of the recording electrode [3]. We here present results from a biophysically detailed computational study of this LFP footprint, focusing on the thalamocortical LFP response in layer 4 of rodent barrel cortex [1]. As illustrated in Fig. 1, the model considers large populations of synaptically activated RS (regular spiking cells) and/or FS (fast-spiking cells) (Fig. 1AB). The computational model, implemented in Python with NEURON, is constrained to predict plausible intracellular EPSCs [4] (Fig. 1C) and EPSPs (Fig. 1D). The model not only predicts the LFP (Fig. 1E), but also the ground-truth CSD (current source-density) (Fig. 1F) that can be used to test CSD estimation methods [5]. Candidate models mimicking experimental findings [1,2] will be presented.

**Figure 1:**
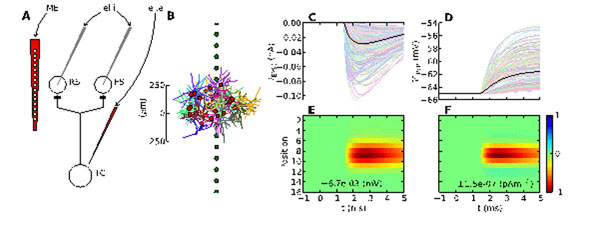
Model overview. **A.** Schematic of recording of unitary thalamic projection pattern to layer 4 with thalamic single-unit electrode (el.e), cortical clamp electrodes (el.i) and cortical multielectrode (ME) [1,2,4]. **B.** Example model population of reconstructed RS cells with ME penetrating population. **C.** EPSCs of model population, black line: average EPSC. **D.** EPSPs of model population, black line: average EPSP. **E.** ME LFP response **F.** ‘Ground truth’ CSD.

## References

[B1] Swadlow HA Gusev AG Bezdudnaya T Activation of a cortical column by a thalamocortical impulse J Neurosci 2002 22 7766 7773 12196600 10.1523/JNEUROSCI.22-17-07766.2002 PMC6757983

[B2] Jin J Wang Y Swadlow HA Alonso JM Population receptive fields of ON and OFF thalamic inputs to an orientation column in visual cortex Nat Neurosci 2011 14 232 238 10.1038/nn.2729 21217765

[B3] Pettersen KH Hagen E Einevoll GT Estimation of population firing rates and current source densities from laminar electrode recordings J Comp Neurosci 2008 24 291 313 10.1007/s10827-007-0056-4 17926125

[B4] Hull C Isaacson JS Scanziani M Postsynaptic mechanisms govern the differential excitation of cortical neurons by thalamic inputs J Neurosci 2009 29 9127 9136 19605650 10.1523/JNEUROSCI.5971-08.2009 PMC2753516

[B5] Pettersen KH Devor A Ulbert I Dale AM Einevoll GT Current-source density estimation based on inversion of electrostatic forward solution: Effects of finite extent of neuronal activity and conductivity discontinuities J Neurosci Methods 2006 154 116 133 10.1016/j.jneumeth.2005.12.005 16436298

